# Hitting the Brakes: Driving Cessation, Cognitive Health, and the Mediating Role of Social Relationships Among Older Adults in the US

**DOI:** 10.1002/alz70858_099940

**Published:** 2025-12-25

**Authors:** Kent Jason Go Cheng, Nelson A. Roque

**Affiliations:** ^1^ The Pennsylvania State University, University Park, PA, USA

## Abstract

**Background:**

This study assesses the association of driving cessation on cognitive health of older adults in the U.S., taking into consideration the potential mediating effect of social relationships.

**Method:**

We used data on older adults aged 65‐85 from the 2010, 2012, 2014, 2016, and 2018 wave of the U.S. Health and Retirement Study (*n* = 12,913 person‐years, 8,769 persons). Outcome was cognitive function based on the Langa‐Weir classification (1=cognitively impaired but no dementia and dementia, 0=normal). Driving cessation (1=yes) was the key predictor, while perceived social support from spouse, children, other family members, and friends were the mediators considered. To formally test mediation, we applied Karlson, Holm, and Breen's (KHB) method to panel logistic regression models. The analyses controlled for age, sex, race, socioeconomic status, type of residence, physical health status, functional limitations, and survey wave.

**Result:**

Driving cessation is associated with a 33.6% increase in the probability of cognitive impairment and dementia. Perceived social support slightly attenuates this association by 1.7%, with friendships accounting for 65.6% of the attenuation. In contrast, support from spouses, children, and other family members combined contributes only half (34.4%) as much to the mitigating effect as friendships.

**Conclusion:**

Driving cessation is associated with an increased risk of cognitive impairment and dementia among older adults, with social support from friends playing the largest role in attenuating this risk. These findings highlight the importance of fostering social connections beyond the family, ensuring access to alternative transportation options, and strengthening broader support systems for older adults to promote cognitive health in aging populations. Future research should investigate the bidirectional relationship between driving cessation and cognitive decline, particularly whether driving cessation reduces mental stimulation or if cognitive changes prompt older adults to stop driving.

